# Type I Gastric Neuroendocrine Tumor Presenting as Acute Upper Gastrointestinal Bleed

**DOI:** 10.7759/cureus.15343

**Published:** 2021-05-31

**Authors:** Anusha Bapatla, Ameena Syed, Abu Fazal Shaik Mohammed, Cortney V Jones, Rana Ismail

**Affiliations:** 1 Internal Medicine, Wayne State University, Detroit Medical Center (DMC) Sinai Grace Hospital, Detroit, USA; 2 Hematology and Oncology, Wayne State University, Detroit Medical Center (DMC) Sinai Grace Hospital, Detroit, USA

**Keywords:** gastric neuroendocrine tumors, chronic atrophic gastritis, pernicious anemia, enterochromaffin like cells, type 1 gastric neuroendocrine tumors

## Abstract

Gastric neuroendocrine tumors (GNETs) are rare and subdivided into type I, type II, and type III. Types I and II are gastrin-dependent and are usually benign, whereas type III is gastrin-independent and more aggressive. Type I accounts for 70-80% of all GNETs. Most of them are asymptomatic and incidentally detected on endoscopy. It can sometimes present with iron and B12 deficiency, dyspepsia, and less commonly with an upper GI bleed.

We present a case of type I GNET who came to the hospital with melena and esophagogastroduodenoscopy (EGD) showing a 3-cm bleeding polyp and histopathology revealing a well-differentiated neuroendocrine tumor with angioinvasion.

## Introduction

Gastric neuroendocrine tumors (GNETs) are rare tumors with a prevalence of 0.17 per 10,000 in the United States [1]. Neuroendocrine tumors (NETs) arise from peripheral neuroendocrine cells present in multiple organs. The most common sites for NETs are the GI tract and lungs. The small intestine accounts for 41.8% of all GI neuroendocrine tumors whereas GNETs constitute only 8.7% of all GI neuroendocrine tumors [2].

Based on etiology, pathogenesis, and histopathological behavior, GNETs are divided into three subtypes - type I, type II, and type III. Both types I and II arise from enterochromaffin-like cells (ECL cells) and are related to hypergastrinemia. The basis for the elevation of gastrin levels in type I is hypochlorhydria secondary to chronic atrophic gastritis (CAG). In type II, the hypochlorohydria is due to Zollinger-Ellison syndrome (ZES) and multiple endocrine neoplasia type 1 (MEN1) [3-5]. Type 1 usually presents with nonspecific symptoms like dyspepsia (70%) and/or anemia (>70 % of patients) and can be incidentally found on endoscopy [6]. These tumors are usually indolent and benign. Even though recurrences are common, the risk of distant metastases is very low [7-9]. Here, we present the case of type I GNET presented with an upper GI bleed.

## Case presentation

A 63-year-old female with a history of hypothyroidism, hypertension, and ductal carcinoma in situ status post right partial mastectomy presented to the hospital with melena of three days. Upon admission, she stated generalized weakness and fatigue for two weeks but did not report nausea, vomiting, abdominal pain, hematochezia, diarrhea, or constipation. The patient had orthostatic hypotension on presentation. The laboratory tests were pertinent for hemoglobin of 6.3 g/dl (no baseline available); mean corpuscular volume of 108.7; red cell distribution width of 30. Vitamin B12 (135 pg/ml), iron (12 µg/dl), and ferritin (23.4 ng/ml) were low with normal folate levels. She received one unit of packed red blood cells, after which repeat hemoglobin increased to 7.4 g/dl. The patient had elevated serum gastrin (1988 pg/ml) and chromogranin A (CgA) levels (300 ng/ml) and normal 5-hydroxyindoleacetic acid (5-HIAA) level in urine (8.4mg/ml). 

**Figure 1:**
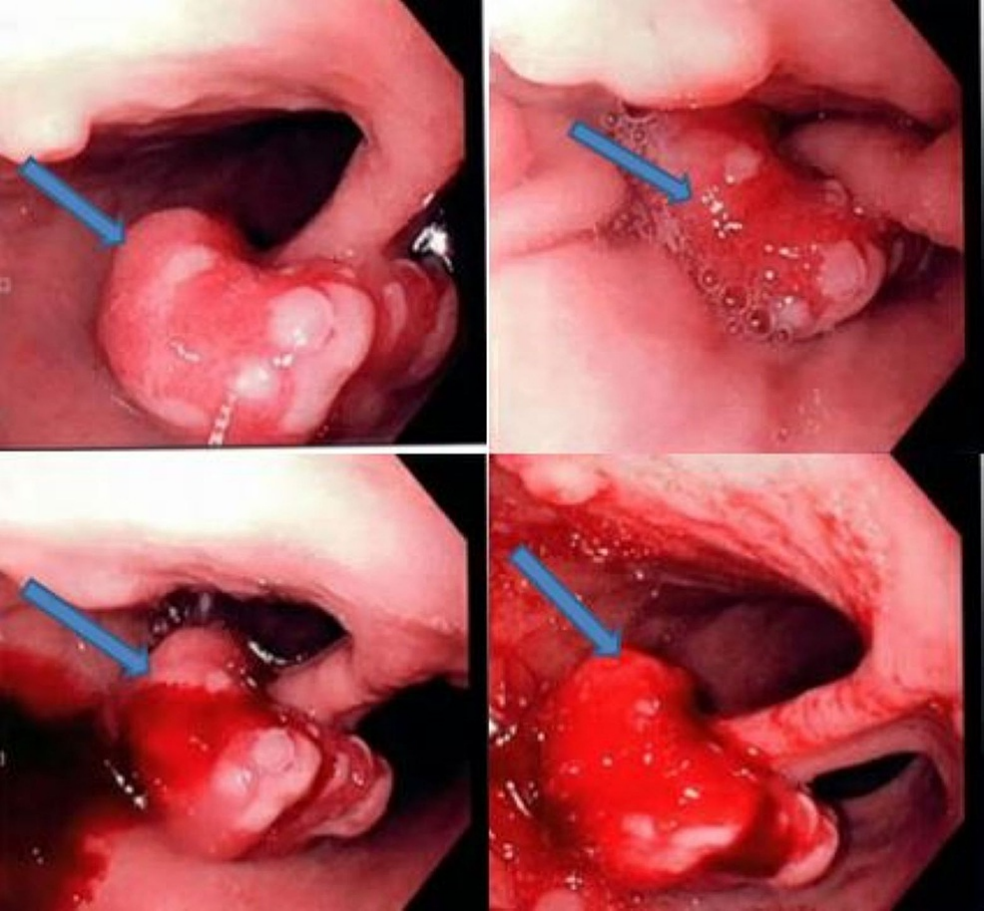
Endoscopy images showing three cm polypoidal, ulcerated, bleeding polyp in the body of the stomach (blue arrow)

Esophagogastroduodenoscopy (EGD) and colonoscopy were performed to localize the source of bleeding. Colonoscopy was unremarkable. EGD showed a 3 cm polypoidal, ulcerated, friable, bleeding mass lesion in the body of the stomach (Figure 1). EGD also showed evidence of atrophic gastritis.

**Figure 2:**
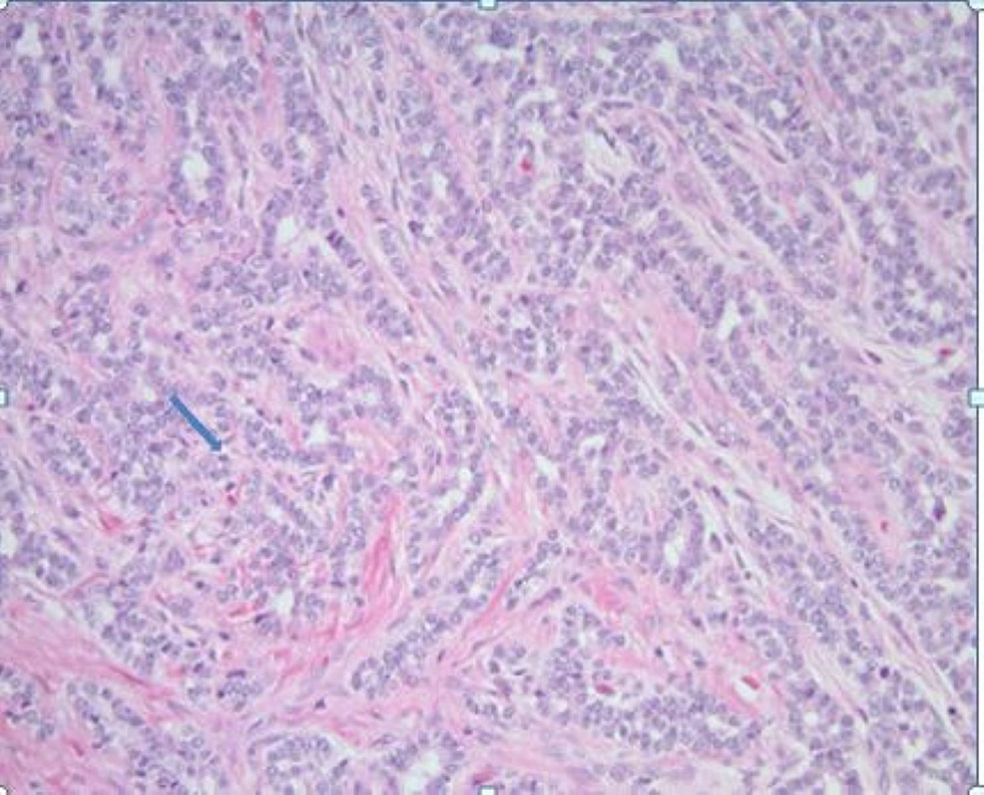
Histopathology images showing neuroendocrine tumor cells (blue arrow)

**Figure 3:**
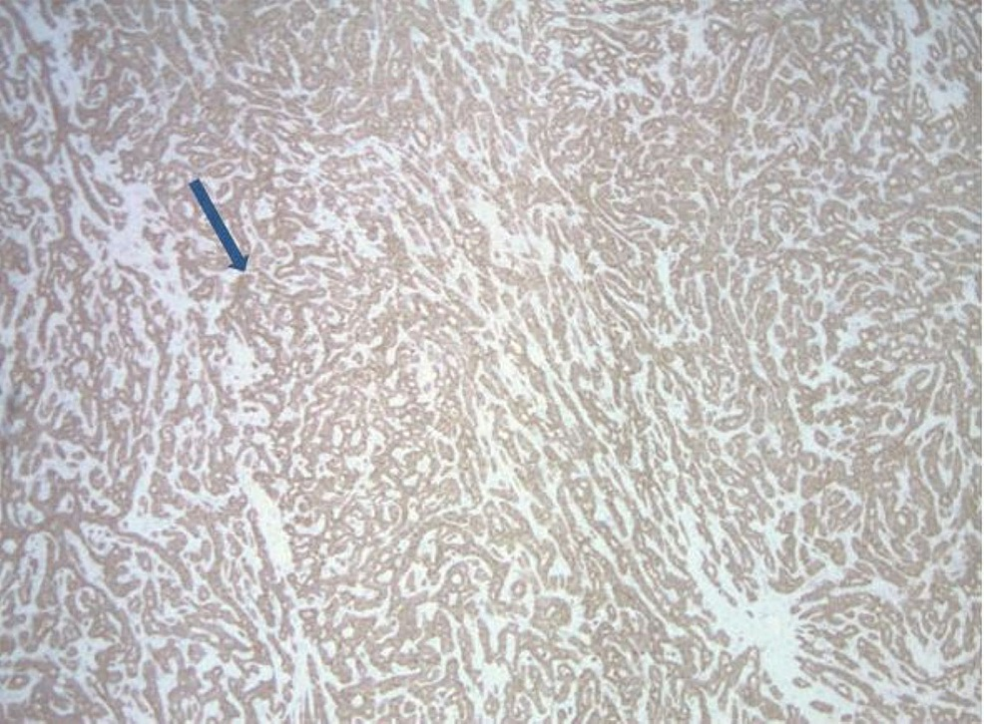
Immunohistochemical staining showing chromogranin-positivity (blue arrow)

**Figure 4:**
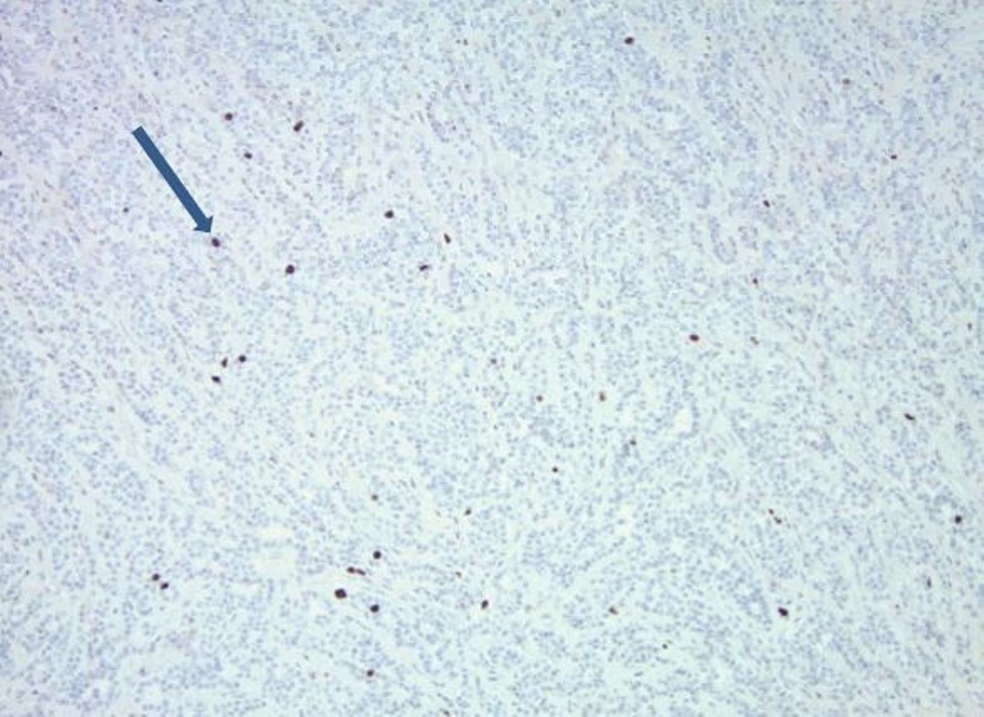
Histopathology images showing Ki 67 activity of less than 3% (blue arrow)

**Figure 5:**
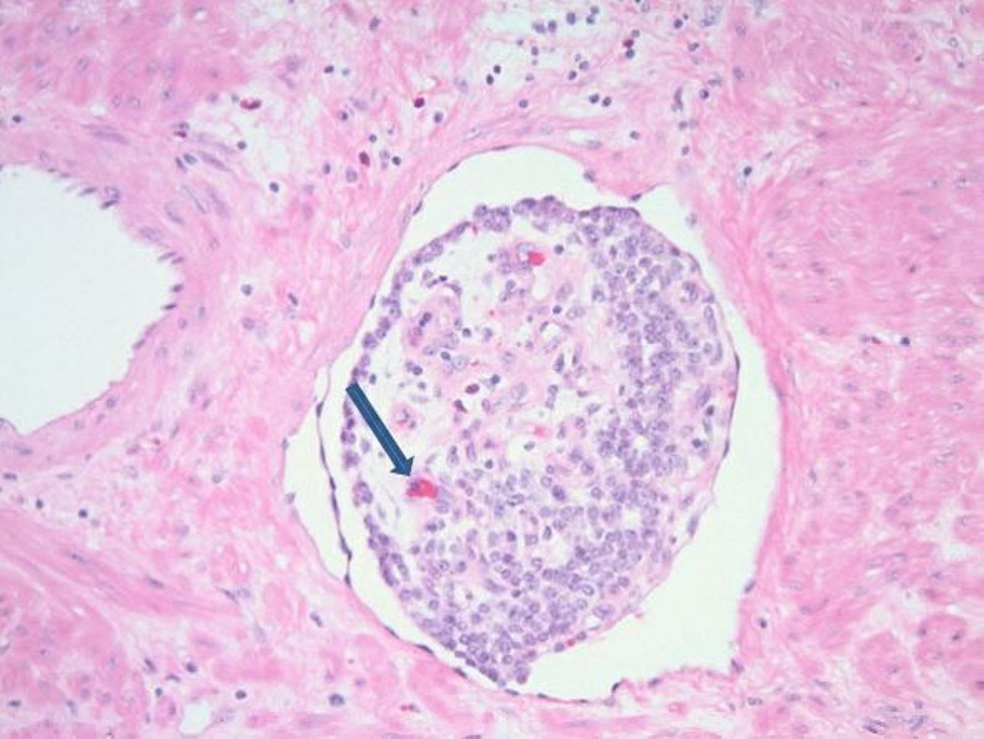
Histopathology images showing angio-invasion of the tumor (blue arrow)

**Figure 6:**
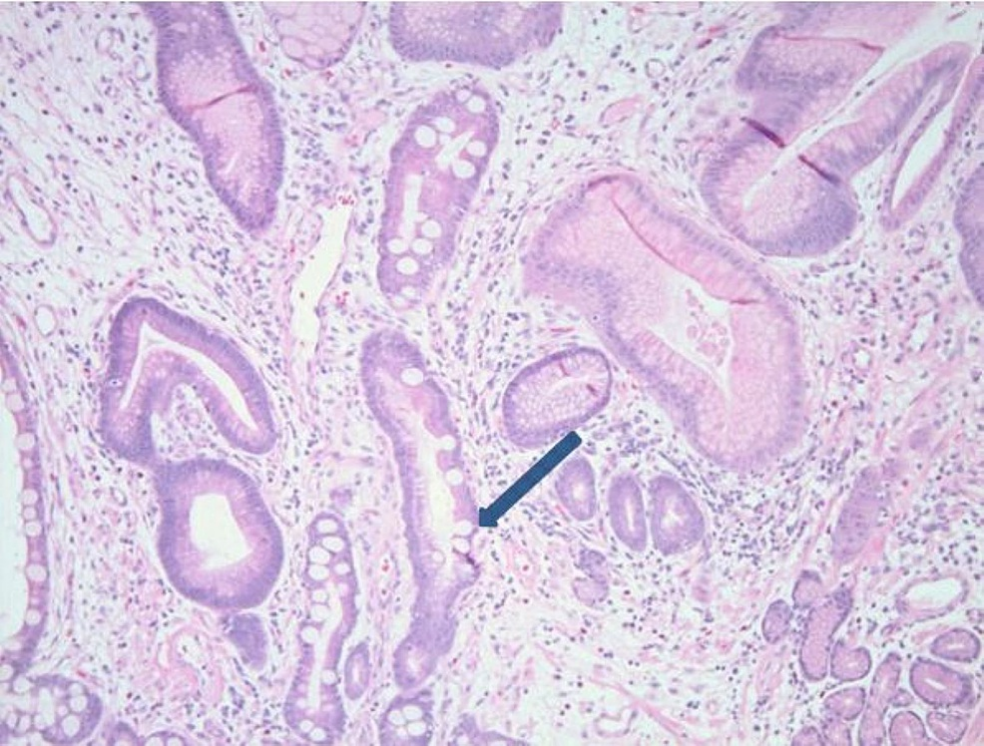
Histopathology images showing intestinal metaplasia (blue arrow)

Snare polypectomy was done, and to control bleeding, two clips were applied, and an epinephrine injection was given. Gross appearance showed a 3.5 x 3.0 x 2.8 cm mass with a 0.9 x 0.8 cm base (deep resection margin). Microscopically, the mass was composed of tumor cells, which were relatively monotonous and arranged in predominantly glandular structures (Figure 2). The tumor cells were diffusely and strongly positive for synaptophysin and CgA on immunohistochemical studies (Figure 3), and the ki-67 proliferative index was less than 3% (Figure 4). This tumor demonstrated angioinvasion but margins are free of tumor cells (Figure 5). Microscopy also showed moderately severe chronic inactive gastritis, prominent foveolar hyperplasia, focal intestinal metaplasia, and superficial erosions (Figure 6). The features above were consistent with grade 1 and well-differentiated neuroendocrine tumors with the background of CAG.

CT scan of thorax and abdomen showed no evidence of metastasis. The patient was discharged on cyanocobalamin 1000 mcg weekly for four weeks and then monthly, with follow-up endoscopy in three months.

## Discussion

Type I GNETs account for 70-80% of GNETs, arise from the ECL cells hyperplasia, and are associated with hypergastrinemia due to CAG. Type II accounts for 5-6% of GNETs and is associated with hypergastrinemia due to Zollinger-Ellison syndrome and MEN-1. In both types I and II GNETs, the arousing factor is hypergastrinemia with different etiology. An increase in gastrin levels causes stimulation of ECL cells, which leads to cellular hyperplasia and dysplasia, resulting in GNETs [3,4,6]. Type III is gastrin-independent and accounts for 20% of GNETs. Types I and II are primarily multiple, small (<1 cm), benign, and involve the mucosa and the submucosa, whereas type III are single, large (>2 cm), more aggressive and associated with local and distant spread, and involve the muscular layer [3,6]. 

Type I GNETs are prevalent in older women often diagnosed in their 60s [1]. They are associated with CAG either due to autoimmune gastritis or H. pylori infection. Other independent risk factors for developing type I GNETs are increased age, intestinal metaplasia, and elevated CgA levels [10]. Autoimmune metaplastic atrophic gastritis increases the risk of type I GNETs by 12 fold [11]. Additionally, type I GNETs are associated with pernicious anemia in almost 50% of patients [11]. They may be associated with other autoimmune diseases like type 1 diabetes mellitus, autoimmune thyroiditis, and primary biliary cirrhosis [3]. Destruction of parietal cells in CAG leads to achlorhydria/hypochlorhydria, which causes stimulation of antral gastrin cells, resulting in hypergastrinemia. High gastrin levels stimulate the ECL cells causing hyperplasia and dysplasia of these cells, which result in type I GNETs [3,4]. 

GNETs typically present with non-specific symptoms and are found incidentally on endoscopy. They may present with iron and vitamin B12 deficiency [12]. Also, they infrequently present with GI bleed [12]. Our patient presented with an upper GI bleed, which is an unusual manifestation for type I GNETs. GNETs can also show features of carcinoid syndrome, which include flushing and tachycardia. However, type I GNETs do not manifest carcinoid features, unlike type III that are commonly associated with hepatic metastasis, hence manifest carcinoid features. In our case, 5-HIAA levels were normal and did not manifest any carcinoid features, as elevated levels tend to associate with more advanced GNETs with poor prognosis [13].

The mainstay of GNETs diagnosis is endoscopy and biopsy. Endoscopically, in about 65% of cases, type I GNETs appear as multiple, small, red, or yellow sessile polypoidal or smooth hemispherical lesions in the gastric fundus or body, involving the mucosa and the submucosa. In about 22% of patients, lesions may be found microscopically for biopsies taken for other reasons [13,14]. When macroscopically visible, each polyp may show a central depression associated with sub-epithelial growth [6]. In our case, endoscopy showed a large, 3 cm, solitary pediculate polyp with profuse bleeding, which is not a typical presentation in type I GNETs. We had to use two clips and an epinephrine injection to stop the bleeding.

Endoscopic ultrasound may help assess the exact depth of the tumor and lymph node metastasis, but its exact clinical usability and diagnostic capability are questionable [4,14]. CT or MRI should be done to monitor any local or metastatic spread, while octreotide scans are helpful to detect local and distant metastasis [5]. Recent studies showed that positive emission tomography (PET) scan with 68Ga labeled is as efficacious as an octreotide scan and is more cost-effective [4].

Biopsy of the polyp should establish the diagnosis of GNET, and biopsy of the non-polypoidal area usually establishes associated CAG [6]. Histology is usually well differentiated. Immunohistochemical staining of NETs is positive for CgA, synaptophysin, somatostatin receptor 2A, and monoamine vesicular transporter [5]. Positive CgA staining indicates ECL hyperplasia and dysplasia. High CgA in patients with CAG is a strong predictor of type I GNET development [8]. 

The European Neuroendocrine Tumor Grading system is usually used to grade GNETs. This system uses mitotic activity in 10 high-power fields and a percentage of the Ki-67 index. Based on this, GNETs are divided into grades 1, 2, and 3. They are classified as grade 1 when mitotic activity is <2 and/or Ki-67 index is less than 3%, grade 2 when mitotic activity is two to 20 and/or Ki-67 percentage is 3-20%, and grade 3 (neuroendocrine carcinoma) when mitotic activity is > 20 and or Ki-67 percentage is > 20% [15]. Our patient had a Ki-67 percentage of less than 3% and rare mitotic activity and was classified accordingly as grade 1 GNET. 

The management options available for type I GNETs include medical management, endoscopic surveillance, or surgical management [1]. The medical management options include somatostatin analogs and gastrin/cholecystokinin 2 {CCK2} inhibitors. Somatostatin analogs inhibit gastrin production and hence decrease ECL cell hyperplasia. Netazepide, a potent gastrin inhibitor, helps decrease the size and number of tumors, but its long-term effects are yet to be studied [1,5]. The type of the tumor is important to guide treatment. Type III is more aggressive and should be managed similarly to gastric adenocarcinoma [4,14]. Types I and II are benign, and their management depends on various factors such as the size, the depth, and the number of polyps. Type 1 GNETs less than 1-2 cm and without muscularis propria involvement can be effectively managed with simple surveillance or endoscopic resection [4]. Noh et al. in their retrospective analysis of 125 patients, showed that endoscopic resection is safe and effective when the size is less than 1-2 cm, with no muscularis propria involvement or no lymph node involvement, and no distant metastasis. Simple surveillance without endoscopic resection was not supported in this study [16]. European consensus guidelines recommend endoscopic resection when size is less than 1 cm [16]. More extensive tumors of more than 1-2 cm are managed with endoscopic resection or surgical resection. The antral gastrectomy is recommended in persistent and recurrent cases since it removes gastric G cells and eventually decreases gastrin production, thus preventing ECL cell hyperplasia and dysplasia [4]. Endoscopic resection procedures include polypectomy, endoscopic mucosal resection, and endoscopic submucosal dissection. Noh et al. showed no difference in mortality and morbidity, although there was complete resection with endoscopic submucosal dissection than endoscopic mucosal resection [16]. Our patient had a tumor size of 3 cm and polypectomy margins are free of tumor and also CT showed no evidence of metastasis. So the plan is for close follow-up without surgical intervention.

Type I GNETs are usually benign, and a minority of patients develop lymph node metastasis, and very few develop distant metastasis. The risk of metastasis is <5% [5]. Even though the recurrences are high with type I GNETs, morbidity and mortality are low, with a five-year disease-specific survival of 100% [16]. Tsolakis et al. suggested tumor size and depth of invasion as the most significant prognostic factors in their systemic review. They also mentioned that the tumor grade has no role in predicting tumor progression [5]. Deep mucosal or angio-lymphatic invasions are also considered one of the poor prognostic factors for metastasis. In their retrospective analysis, Panzuto et al. showed that grading does not significantly correlate with poor outcomes. They also found that age, tumor size, and tumor type are independent poor prognostic factors [17]. Our patient demonstrated angioinvasion, one of the poor prognostic factors which require closer follow-up after endoscopic resection.

## Conclusions

Type I GNETs can present with a wide variety of symptoms, including upper GI bleed. With an increase in the incidence of GNETs, physicians should be aware of the different presentations of GNETs. For tumors more than 1 cm, exact factors to decide for surgical resection or endoscopic resection were not clearly proposed. So, we are recommending further studies to establish exact guidelines regarding management and follow-up of type I GNETs more than 1 cm while considering all the poor prognostic factors.
